# An RNA-centric historical narrative around the Protein Data Bank

**DOI:** 10.1016/j.jbc.2021.100555

**Published:** 2021-03-18

**Authors:** Eric Westhof, Neocles B. Leontis

**Affiliations:** 1 Institut de Biologie Moléculaire et Cellulaire du CNRS, Architecture et Réactivité de l’ARN, Université de Strasbourg, Strasbourg, France; 2 Department of Chemistry, Bowling Green State University, Bowling Green, Ohio, USA

**Keywords:** RNA, structural biology, databases, Protein Data Bank, modeling, computational biology, PDB, Protein Data Bank, PDBj, PDB Japan, PDP, Programmed Data Processor, NDB, Nucleic Acid Database, RNP, RNA–protein complex, wwPDB, worldwide PDB

## Abstract

Some of the amazing contributions brought to the scientific community by the Protein Data Bank (PDB) are described. The focus is on nucleic acid structures with a bias toward RNA. The evolution and key roles in science of the PDB and other structural databases for nucleic acids illustrate how small initial ideas can become huge and indispensable resources with the unflinching willingness of scientists to cooperate globally. The progress in the understanding of the molecular interactions driving RNA architectures followed the rapid increase in RNA structures in the PDB. That increase was consecutive to improvements in chemical synthesis and purification of RNA molecules, as well as in biophysical methods for structure determination and computer technology. The RNA modeling efforts from the early beginnings are also described together with their links to the state of structural knowledge and technological development. Structures of RNA and of its assemblies are physical objects, which, together with genomic data, allow us to integrate present-day biological functions and the historical evolution in all living species on earth.

The *Protein Data Bank* (PDB) is an icon for structural biologists. In its virtual vaults, the PDB stores the structures of biological macromolecules obtained painstakingly by thousands of crystallographers, electron microscopists, and nuclear magnetic resonance spectroscopists over the last 50 years or so. This note is primarily written to honor and acknowledge those who set up the PDB and who have strived throughout all these years to improve and maintain the database. We thank the *Journal of Biological Chemistry* for organizing the publication of such pieces that provide a mixture of personal recollections, historical records about nucleic acids structure, and some general comments about databases and their future.

## The evolution of structural databases

When one of us was a postdoctoral fellow with M. Sundaralingam at the Department of Biochemistry of the University of Wisconsin in Madison, the only way to work with a published molecular structure was to first type in the atomic coordinates at the console of a PDP computer (Programmed Data Processor from Digital Equipment Corporation, Massachusetts). Only then could one deduce or check atomic distances, angles, stereochemistry, contacts and visualize the 3D structure using programs developed in-house. This was a tedious job, but very instructive to students and postdoctoral fellows. In those days, atomic coordinates were part of the paper and presented with temperature factors in tables (for an example, see (1) and for general guidelines (2)). The number and diversity of errors that escaped detection before publication, while rarely casting doubt on the crystallography itself, led generally to inaccurately described molecular structures with, for example, some wrong chiralities, or atoms of the molecular unit in different unit cells. This manual handling of data constituted for sure an excellent training ground. Happily, the nucleic acid structures that could be tackled at that time were restricted to the bases (often modified), nucleosides, and nucleotides, with the first structure of a dinucleotide appearing in 1971 (3, 4). In 1965, Olga Kennard, inspired by J.D. Bernal, had started to work on the *Cambridge Structural Database*, CSD (5) and, in 1971, the *PDB* was established jointly by the *Crystallographic Data Centre*, Cambridge and the *Brookhaven National Laboratory* (Protein Data Bank). PDB originally served as a repository system where crystallographers could mail in their data on punch cards, then computer tapes, for archiving and distribution. The announcement of the founding of the PDB in *Nature New Biology* was a simple little insert of 256 words (6)! Full publications appeared later (6, 7). The CSD archived structures of individual nucleotides, as well as di- and trinucleotides. These structures are not in PDB. In fact, the first release of PDB did not include any nucleic acid structures, just seven protein structures (7). Later, in 1992, the Nucleic Acid Database (NDB) was founded by Berman *et al.* (8) and structures of RNA units were included in the NDB.

In the early days of the development of structural databases, the main objectives were to prevent the loss of data by archiving solved structures and making them freely available upon request. The deposited data were checked for atom names, numberings, and geometry by database curators, but not as systematically and thoroughly as it is done today. Dictionaries and defined formats were developed over the years and applied to standardize nomenclature, file formats, and metadata. As regards nucleic acids, the relational NDB archived data accompanied by structural information and descriptions (8). NDB served as a testbed for new data formats that could accommodate much larger structures and much more metadata, which were eventually adopted by PDB. Nowadays, these databases are rich in metadata and tools for assessing and describing structures. Very valuable tools for structural validations (9) and corrections (10) of nucleic acid structures were introduced. The PDB now offers various validation tools with compelling metrics for all types of structures (11). While the PDB remains, understandably, protein-centric, other databases and tools are available for nucleic acids (8, 12, 13)—see also https://www.bgsu.edu/research/rna/.

## Quality and accuracy of structural data

All of the precise structural data regarding RNA comes ultimately from atomic-resolution X-ray structures of nucleotides, oligonucleotides, and various biologically relevant structures, ranging in size from individual helical elements to the full ribosome. The early work was carried out by pioneers such as A. Rich, O. Kennard, and M. Sundaralingam, who from the mid-1960s and into the 1980s carried out precise crystallographic studies of nucleosides, nucleotides, and dinucleotides. These data comprise all our basic knowledge of bond lengths, angles, and stereochemistry, as well as interaction preferences, including all types of base pairs, and most stacking and base–backbone interactions. The new data led early on to useful concepts including the conformational wheel, describing ribose conformations (14), and better understanding of the conformational preferences of nucleic acids, the analysis of which was quite daunting to theoreticians and modelers alike (15, 16, 17). Unlike proteins, which effectively have just two degrees of freedom per monomer unit, nucleotides present seven, six dihedrals along the backbone, and the one around the glycosidic bond. In 1965, based on the small number of protein structures known, as well as better resolved peptide structures, Ramakrishnan and Ramachandran (18) worked out the sterically favored backbone configurations and summarized them graphically in two dimensions, the dihedral angles phi and psi. Such Ramachandran plots continue to be used as easily visualized metrics for protein structures. Shortly afterward, Sundaralingam (19) carried out a series of landmark studies to accurately determine molecular structures of nucleic acid constituents, culminating in a highly cited 1969 paper in *Biopolymers* that laid the foundation for conformational analysis of nucleotides and polynucleotides, and gave the first stereochemical rules for the sugar-phosphate backbone. This work revealed conclusively that backbone dihedrals are restricted and that correlations exist between them and supported theoretical work that proposed various schemes to simplify nucleic acid conformational analysis using virtual bonds (15, 20).

High-resolution data from small molecules are also used to build force fields and to infer rules for assembly of molecular moieties. These force fields and energetic rules are then used for producing and optimizing structures, sampling the conformational space, or simulating molecular dynamics. The quality and general value of the deduced force fields strongly depend on the number and variety of structures available. The importance of efficient and accurate force fields is paramount in modern biochemical research, since these force fields are used not only for computing trajectories of molecular dynamics simulations, but also in determination of new structure using NMR and cryo-electron microscopy.

It cannot be overemphasized that the quality of the deposited structures is of primary importance; it is directly related to the crystallographic resolution of the X-ray data and on the refinement process, since only a minor fraction of X-ray structures is obtained at true atomic resolution (better than 1 Å). For example, can one really be confident in RNA structures at resolutions above 3.3 Å with average B-factors around 200 Å^2^, bad clash scores, and poor PDB validation metrics? Only a systematic analysis of the various regions of the RNA molecule in electron density maps would allow a knowledgeable and interested person to reach an informed opinion. One key parameter for compiling reference databases for knowledge extraction is the nonredundancy of the structures that are included. Nonredundant structure databases, by reducing bias in the parameters deduced from structures, are extremely valuable for extracting knowledge about RNA as well as for benchmarking modeling strategies (21). In this respect, it is worth noting that less than 100 nonredundant RNA structures have been solved at 2 Å resolution or better. Well-defined metrics for 3D structures determined from crystallography (X-ray and NMR) have been established and are reported at the nucleotide level by PDB (22, 23, 24). Metrics have recently also been defined for structures determined by cryo-electron microscopy, which is largely replacing X-ray diffraction for structure determination of large macromolecular machines such as ribosomes, viruses, and spliceosomes (25).

## The first 20 years of RNA structures

The first nucleic acid structures were of short synthetic DNA helices and were deposited in 1981 (Z-DNA, 2DCG (26) and B-DNA, 1BNA (27)), with the molecular descriptions published respectively in 1979 (28) and 1980 (29). These high-resolution DNA structures only became possible once solid-state synthesis could provide sufficient quantities of pure oligonucleotides for crystallization and structure determination (30, 31). The early RNA structures, of biological origin, were those of various tRNAs isolated from *Escherichia coli* or yeast (32, 33, 34), an accomplishment that required novel purification and crystallization protocols. It took roughly 10 years to produce refined structures of yeast tRNA^Phe^ (deposited in 1978, 4TNA (35) and 6TNA (36)). The choice of tRNA for study was obvious because of the amount present in cells and the availability of some purification protocols. Its primary and secondary structures had been determined in the 1960s, by chemical and biochemical means (37, 38). In the 1980s, additional tRNA structures were solved as well as the first RNA viruses (1BMV (39) and 2TMV (40)), containing short fragments of the viral genome bound to the coat proteins. In those years, at crystallography meetings and conferences, talks on nucleic acids, and RNA in particular, were generally relegated to the last day, either after the meeting dinner (always very lively and joyous events) in the early morning or just before the departure of the bus.

Following novelty and progress in chemical synthesis and purification of RNA oligonucleotides (41), the first X-ray structures of synthetic RNAs appeared in 1988 (42) and were deposited in 1991. That structure showed first examples of intermolecular interactions involving the ribose hydroxyl groups between RNA helices (43). The next advances came from NMR structures of small recurrent RNA motifs, such as the frequent GNRA tetraloop (44) - identified by sequence comparisons in large RNAs (autocatalytic introns, ribosomal RNAs, RNase P). These included most revealingly the loop E of 5S rRNA (45, 46). Even though they lacked atomic resolution, the NMR structures were sufficiently detailed to provide models that expanded our understanding on how the RNA structures could form, in particular the variety of non-Watson–Crick base pairs, and they provided enough information to infer sequence signatures to identify recurrences of the same motifs in other structures (44, 47). The willingness of its directors to expand PDB to include NMR structures proved to be a wise decision.

**Figure 1:**
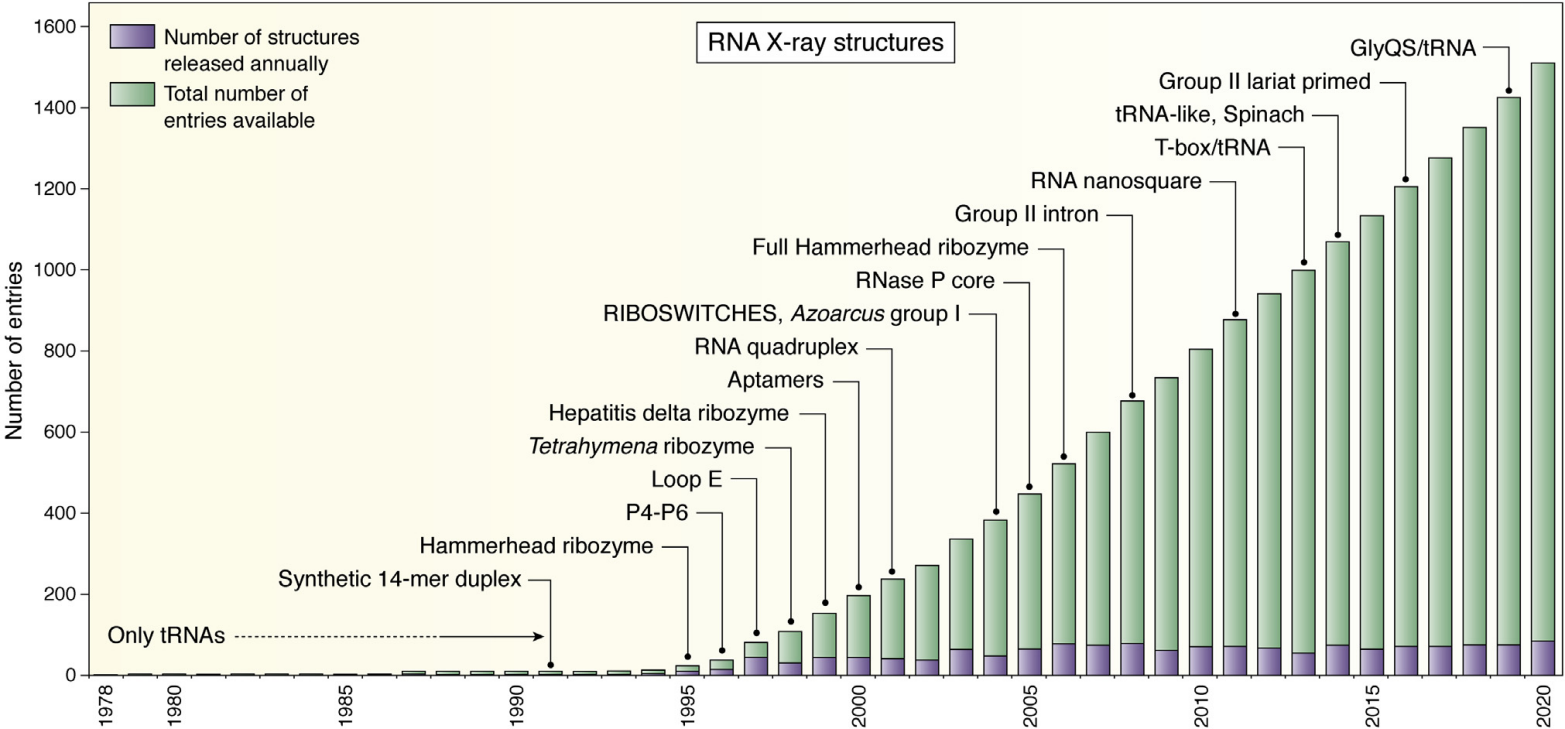
**The evolution of the number of RNA structures in the PDB.** The figure is downloaded from the option “Analyze PDB statistics.” All RNA structures are included (from X-ray, NMR, and cryo-EM). Some key X-ray structures are indicated. Up to 1991, only tRNA structures were present. Following time, these structures are highlighted: (1) A synthetic 14-mer duplex, 1RNA (43); (2) the core hammerhead ribozyme, 1MME (189); (3) the P4-P6 domain of the *Tetrahymena* ribozyme, 1GID (65); (4) the eukaryotic loop E structure, 354D (191); (5) the core of the *Tetrahymena* ribozyme, 1GRZ (200); (6) the hepatitis delta ribozyme, 1DRZ (192); (7) Aptamers binding to malachite green, 1F1T (201), and vitamin B12, 1DDY (202); (8) RNA quadruplex, 1J8G (203); an earlier NMR structure was solved before, 1RAU (204); (9) the purine riboswitch, 1Y27 (119), since then the structures of a great variety of riboswitches have appeared (117, 120, 205); (10) the group I intron from Azoarcus, 1U6B (206); (11) the core of a RNase P ribozyme, 2A2E (207); (12) the full hammerhead ribozyme with long-range loop–loop contacts stabilizing the core, 3ZD5 (208); (13) a complete group II intron, 3EOH (101); (14) the structure of a RNA nanosquare, 3P59 (209); (15) the complex between the T-box riboswitch and its tRNA target, 4MGN (210); (16) the TYMV tRNA-like, 4P5J (211); (17) the Spinach fluorescent aptamer, 4TS0 (113); (18) a group II intron with a lariat primed for transposition, 5J01 (102); (19) the full structure of the T-box between GlyQS and its tRNA, 6POM (212).

**Figure 2:**
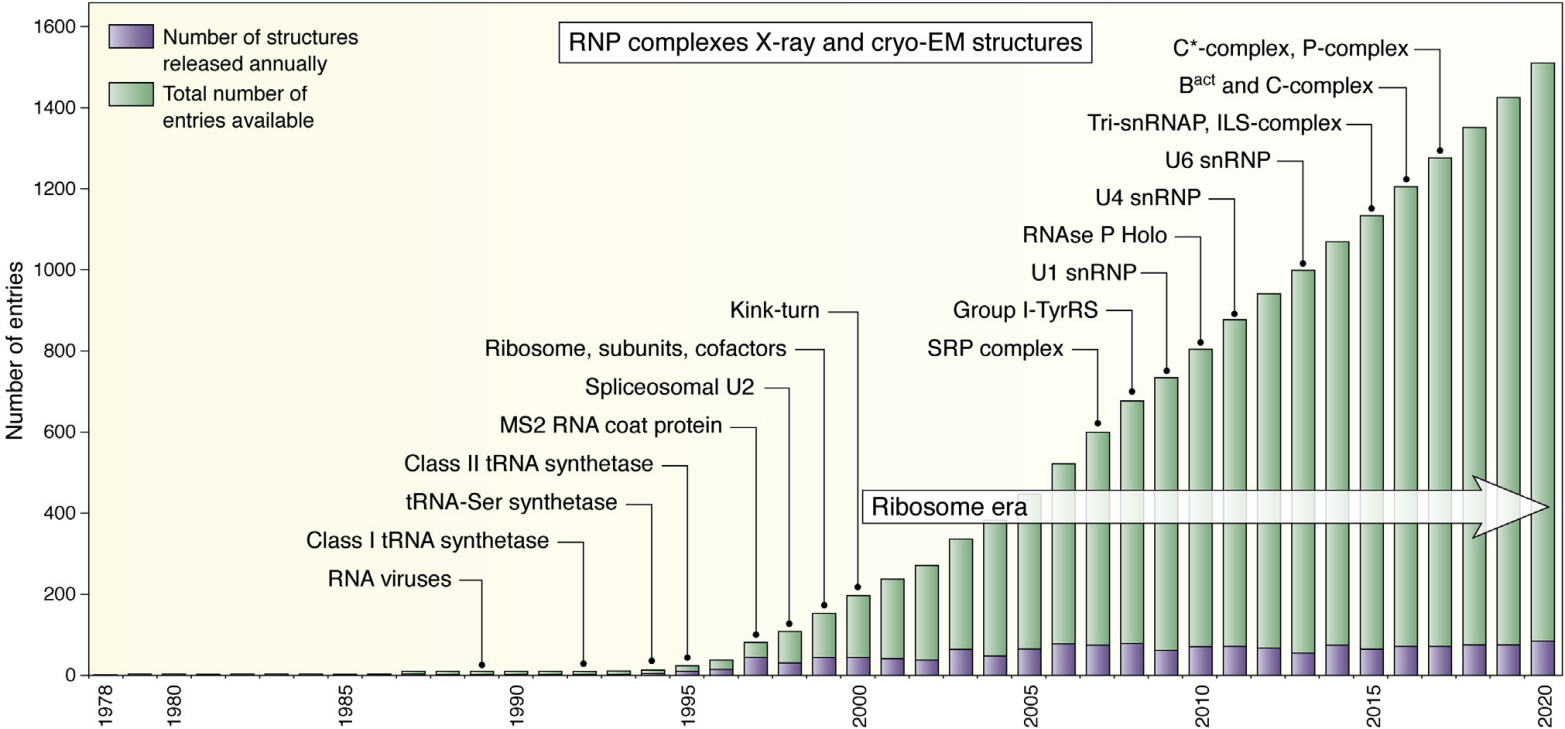
**The evolution of the number of structures of RNA–protein complexes (RNPs) in the PDB.** The figure is downloaded from the option “Analyze PDB statistics.” All RNP structures are included (from X-ray, NMR, and cryo-EM). The number of structures related to ribosomes and its cofactors is much too large to show them on such a figure. We preferred to emphasize the complexes formed in the spliceosome (for detailed reviews, see (99, 213)). Most of the large RNP structures after 2015 are based on cryo-EM data. Following time, these structures are highlighted: (1) RNA viruses, 1BMV (39); (2) class I tRNA synthetase complex, 1GSG (214); (3) tRNA^Ser^, a class II tRNA with a long variable loop, complexed with its specific synthetase, 1SER (215); (4) class II tRNA synthetase complex, 1ASY (216); (5) MS2 RNA coat protein, 1AQ3 (217); (6) spliceosomal U2 complex, 1A9N (218); (7) the kink-turn was first observed in the complex of U4 sRNA fragment, 1E7K (219), before being recurrently observed in the ribosome structure (193); (8) the Signal Recognition Particle complex, 2V3C (220); (9) complex between a tyrosyl tRNA synthetase and a group I intron, 2RKJ (221); (10) U1 snRNP, 3CW1 (222); (11) an RNAse P holoenzyme, 3Q1Q (223); (12) in the U4 snRNP, 4WZJ (224); (13) Lsm/U6 snRNP complex, 4M7A (225); (14) the tri-snRNP structure, 3JCM (226); (15) Intron-lariat complex, 3JB9 (227); (16) B^act^ complex, 5GM6 (228), C-complex, 5GMK (229), 5LJ3 (230); (17) C∗-complex, 5WSG (231), 5MPS (232); P-complex, 5YLZ (233), 6EXN (234), 6BK8 (235).

By 1995, the number of X-ray RNA structures (alone or in complex with protein) in the *PDB* amounted to only about 1% of the present content of total RNA and RNA–protein complex (RNP) structures. Figures 1 and 2 show the evolution in the number of structures of RNA alone and RNP with time. Some key structures are indicated to illustrate the increase in complexity of the solved structures. Again progress in chemical synthesis (48, 49), biochemical RNA production (50) and purification (51), as well as in X-ray technology, including the spreading use of synchrotron radiation at cryogenic temperatures (52, 53) were central to the rapid increase in the resolution of RNA and RNP structures. Many advances in crystallogenesis (54, 55) and in biochemical preparation for crystallization of RNA (56, 57, 58) or RNA complexes (59, 60, 61) appeared in those years and the whole community benefited greatly from those novel approaches. Amazing breakthroughs in cryo-electron microscopy are now accelerating the pace at which highly complex particles can be observed (62, 63, 64).

In 1996, a breakthrough in RNA crystallography was achieved in the laboratories of Tom Cech and Jennifer Doudna with the crystal structure of the P4–P6 domain of the *Tetrahymena* group I intron, a structure twice the size of tRNA, featuring striking compact folds and novel types of RNA tertiary contacts (65, 66). With the amazing structure of P4–P6, many interested scientists learned that large RNAs could also be crystallized starting from *in vitro* synthesis and production. This breakthrough spurred fruitful efforts that quickly expanded our knowledge of the repertoire of surprising and beautiful RNA architectures, culminating in the structures of the ribosomal subunits themselves (67, 68, 69, 70) and the Nobel Prize in Chemistry to Venki Ramakrishnan, Tom Steitz, Ada Yonath in 2009 (https://www.nobelprize.org/prizes/chemistry/). And, after the Nobel Prize in Physiology or Medicine for RNA interference (RNAi) in 2006 to Andrew Fire and Craig Mello, the RNA community is celebrating the 2020 Nobel Prize in Chemistry awarded to Jennifer Doudna and Emmanuelle Charpentier for the structure-based design of the splendidly efficient RNA-programmed Crispr-cas9 system (71, 72).

## The unfolding of RNA structural folding rules

**Figure 3:**
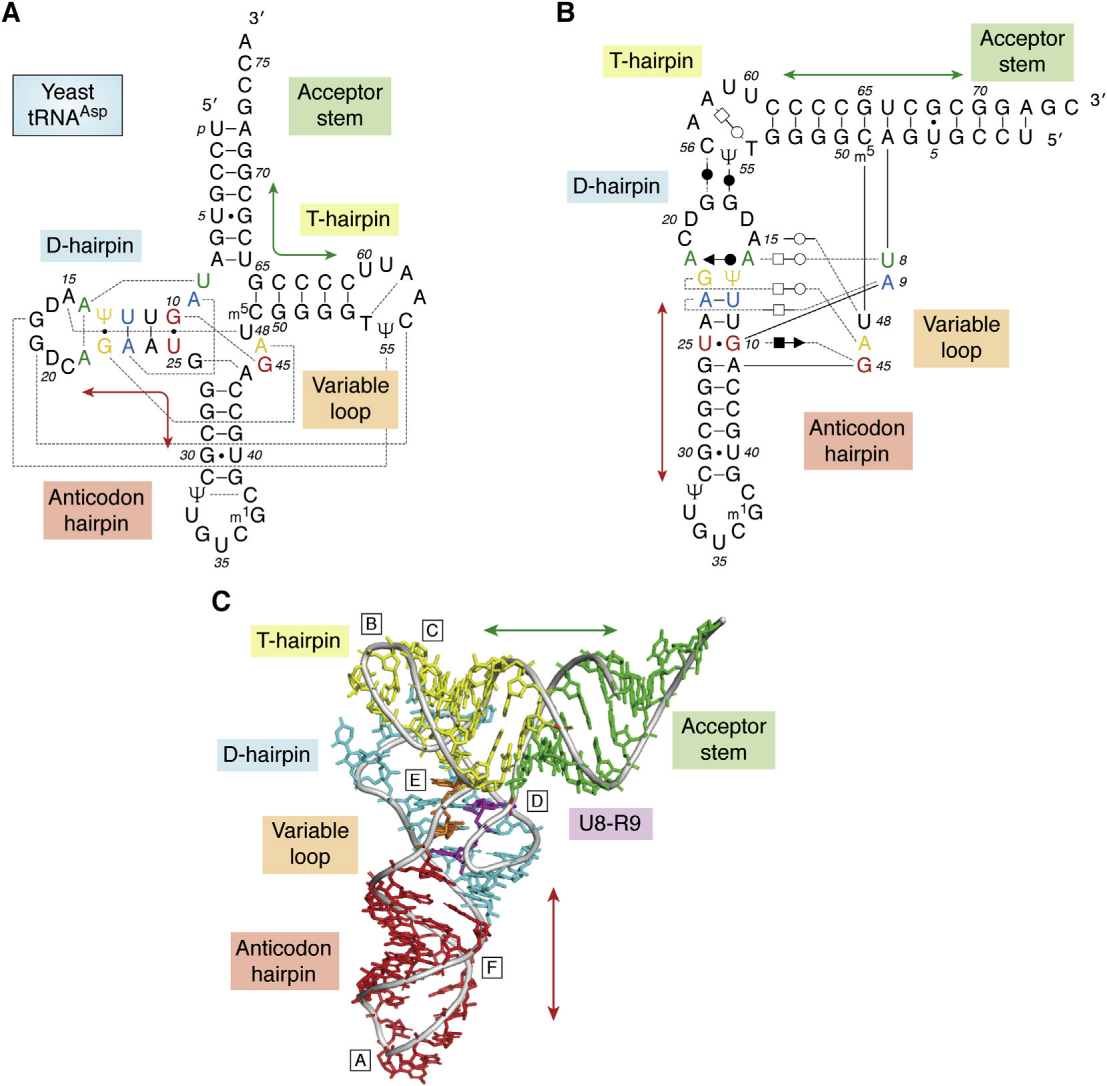
**Three representations of the interactions present between nucleotides in transfer RNA with increasing levels of structural complexity.***A,* standard cloverleaf structure of yeast tRNA^Asp^ (236)**.***B*, a two-dimensional view of the tertiary structure of yeast tRNA^Asp^, it follows the representation proposed by Kim (237) that stresses the two main arms made of helical stems, the acceptor-stem with the Thymine (T)-stem and of the Dihydrouridine (D)-stem with the anticodon stem. A stem capped by a loop is called a hairpin. The numbering follows that of yeast tRNA^Phe^ and because the numbers of nucleotides are not the same in the D- and variable-loops, residues 17 and 47 are skipped and the residue following D20 is C20a. The representation clearly shows the contacts linking the T- and D-loops and the tertiary base pairs and triples between the single-stranded segments and the D-hairpin. The contacts represented correspond to those observed in the yeast tRNA^Asp^ structure (238, 239). For characterizing the tertiary pairs, the following nomenclature is used (240). Nucleic acid bases can interact through three possible edges: the Watson–Crick edge, the Hoogsteen edge (the edge with N7 in purines or C5 in pyrimidines), and the sugar edge (O2 in pyridines or N3 and N2 in purines, with often the hydroxyl O2’ of the ribose). The nucleotides can interact with the sugars on the same side of the H-bonds (like in normal Watson–Crick pairs) and the pair is called *cis*; or on opposite sides and the pair is called *trans*. The three symbols, *circle*, *square*, *triangle*, represent respectively the Watson–Crick, the Hoogsteen, and the sugar edges. When the pair is *cis*, the symbols are *dark* and, when in *trans*, they are *white*. This nomenclature applies to the large number of specific base–base interactions. Pairs form through single H-bond (see Fig. 4*F*) or bifurcated H-bonds (see Fig. 4*B*) are not easily annotated. *C*, the tertiary structure of yeast tRNA^Asp^ with the four domains colored (*green*: acceptor stem; *yellow*; T-hairpin; *blue*: D-hairpin; *red*; anticodon hairpin; the two nucleotides U8 and R9 (generally a purine) linking the 5’-end acceptor strand to the D-strand is *magenta*; the variable loop linking the 3’-end of the anticodon hairpin to the 5’-end T-strand is *orange*). The *double arrows* (*green* and *red*) indicate the sets of helices that stack upon each other in a coaxial manner in the three-dimensional fold. Capital letters indicate the position of the contacts shown in Figure 4.

**Figure 4:**
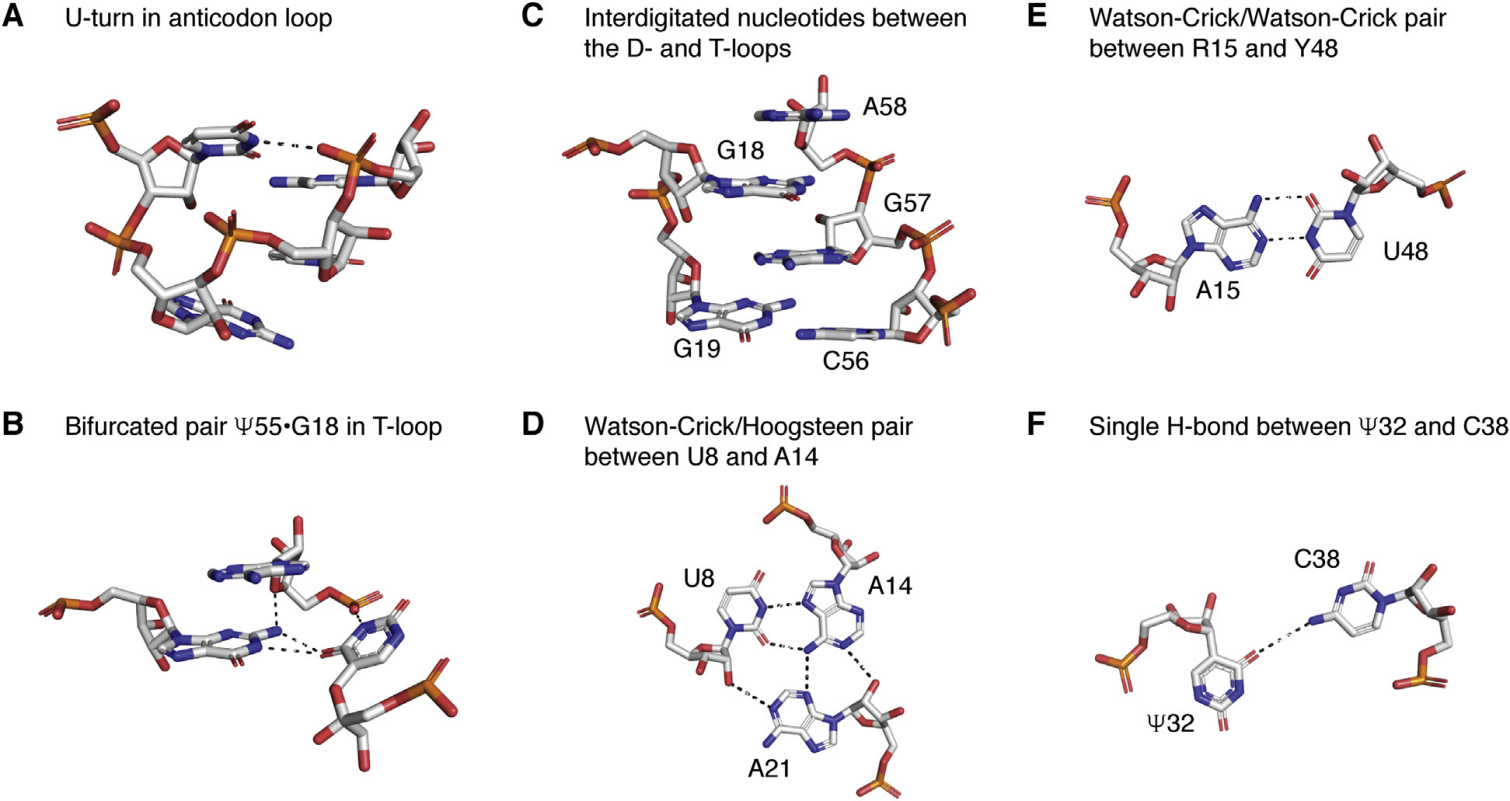
**Illustrations of contacts discussed in the text.***A*, the U-turn after U33 in the anticodon loop: the torsion angle about P-O5’ of the 5’-phosphate of residue 34 is *trans* (180°) instead of the usual *gauche-minus* (−60°); the 5’-phosphate of residue 35 stacks below U33; there is a H-bond between N3-H of U33 and an anionic phosphate oxygen of the 5’phosphate of residue 36. Thus, all three residues of the anticodon triplet have some interaction with the highly conserved U33 (in mammalian initiator tRNA^Met^, C33 occurs). *B*, the equivalent U-turn in the T-loop where the U is a pseudouridine (noted Ψ or Psi). The bifurcated pair Ψ55oG18 in which the O4(Ψ) interacts with both N1-H and N2-H of the guanine is also shown. The residue A58 stacks above G18. *C*, interdigitated nucleotides between the D- and T-loops. *D*, the highly conserved *trans* Watson–Crick/Hoogsteen pair between U8 and A14 forms three H-bonds with A21, also highly conserved. *E*, the famous *trans* Watson–Crick/Watson–Crick pair between R15 and Y48. Levitt (141) noted that residue 15 is always a purine and residue always a pyrimidine and modeled that pair as a regular *cis* Watson–Crick/Watson–Crick. Notice that, in standard nucleotide conformations, *trans* base pairs lead to parallel strands and not antiparallel strands as in usual helices (241). *F*, nucleotides 32 and 38 immediately adjacent to the last base pair of the anticodon stem generally present a single H-bond (for details (242)).

The accumulation of RNA structures, each one bringing either a new insight key to folding or additional confirmation and sequence variants, led to deep understanding of the main physicochemical features underlying RNA architecture. Some of these features, gained from structures of Figure 1, are gathered in Table 1. Up to the 1970s, structures were restricted to the bases, nucleosides, or nucleotides and analyzed the stereochemistry of nucleic acids (19), the orientation of the base with respect to the ribose, stacking (73), sugar pucker (14), protonation states, tautomeric forms, effects of modifications (74). These fundamental studies are still very relevant as shown by the roles of (de)protonation in nucleolytic ribozymes (75, 76, 77) or of tautomeric forms in ribosomal translation (78, 79, 80, 81). Despite its relatively small size (<80 nts), tRNAs have a tightly folded structure and provided many surprising and eye-opening insights into the logic of RNA folding (see also Table 1): short Watson–Crick paired helices coaxially stacked (Fig. 3, *A* and *B*), short-range and long-range interactions (Fig. 3*C*), the central roles of non-Watson–Crick base pairs in mediating tertiary contacts (Fig. 4), base–phosphate or sugar–phosphate contacts (Fig. 4, *A* and *B*), structured hairpin loops (with the famous U-turn) and their propensities to intimately interact, and base–base intercalative stacking interactions (Fig. 4*C*) that contribute to long-range loop–loop interactions.

**Table 1:** A short historical overview of folding rules derived from crystal structures

Date	RNA	Key advances in revealing interactions within RNA structures
50s, 60s, 70s	Bases, nucleo-sides/-tides	Base pairing (74), stacking (73), sugar puckers (14), protonation, tautomers, modifications, metal binding (187)
1976	tRNAs	- coaxial stacking between helices (continuous strand) - loop–loop interactions - non-Watson–Crick base pairs - U-turn structuration of loops - base triple contacts between a single-strand and the deep major groove of a helix - intercalation of unpaired bases - polyamines, Mg ions, lead cleavage
1991	RNA helices	O2’H…O2’ H-bonds between adjacent helices (43), 4-way junctions (188)
1995	Hammerhead ribozyme	3-way junction structured by non-Watson–Crick pairs (189, 190), GNRA/helix minor groove contacts (190)
1996	P4-P6	A-minor contacts, ribose zippers, platform triples, packing of helices, loop–loop interactions (65, 66)
1997	Loop E	Continuous stack of non-Watson–Crick pairs, Mg ions (191)
1999	Hepatitis delta virus ribozyme	Highly constrained pseudoknot, continuous A-minor contacts (192)
2000	K-turn	Recurrent RNA modular unit based on non-WC pairs (193, 194)
2000	Ribosome	A goldmine of molecular contacts. Prevalence of A-minor contacts (193); A-minor recognition of codon/anticodon triplet (194), base–phosphate contacts (195), conservation of GoU pairs (196), multiple junctions (197)

A GNRA tetraloop is a stretch of four nucleotides capping a hairpin and starting with a guanine (G), any of the four nucleotides (A, G, C, U, or N), a purine (A or G, R) and ending with an adenine (A). The GoU pairs were first suggested by Francis Crick (198) for explaining the degeneracy of the genetic code. In such a pair, a U pairs with a G instead of a C. GoU pairs have definite characteristics and play key roles in RNA structures and biology (for a recent overview, see (199)).

**Figure 5:**
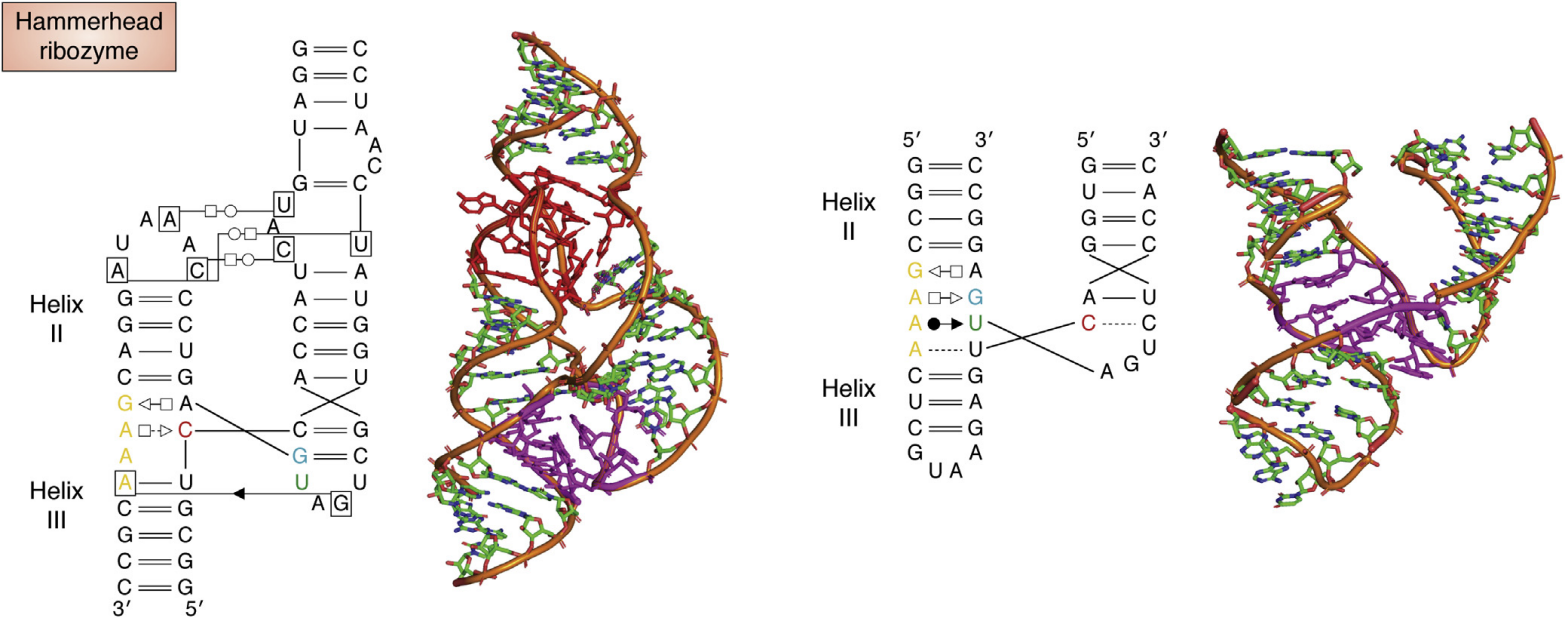
**A native state of the hammerhead ribozyme (*left drawing*) that promotes cleavage at low magnesium concentrations** (243) **that is reached through intricate contacts (and variable depending on the type of ribozymes) between an apical loop and an internal loop (drawn in *red color*).** Without these tertiary contacts, a different core of the three-way junction (drawn in *magenta* in both structures) is observed (compare the regions in *magenta color*). The nomenclature described in Figure 3*B* is used for the non-Watson–Crick pairs (240).

**Figure 6:**
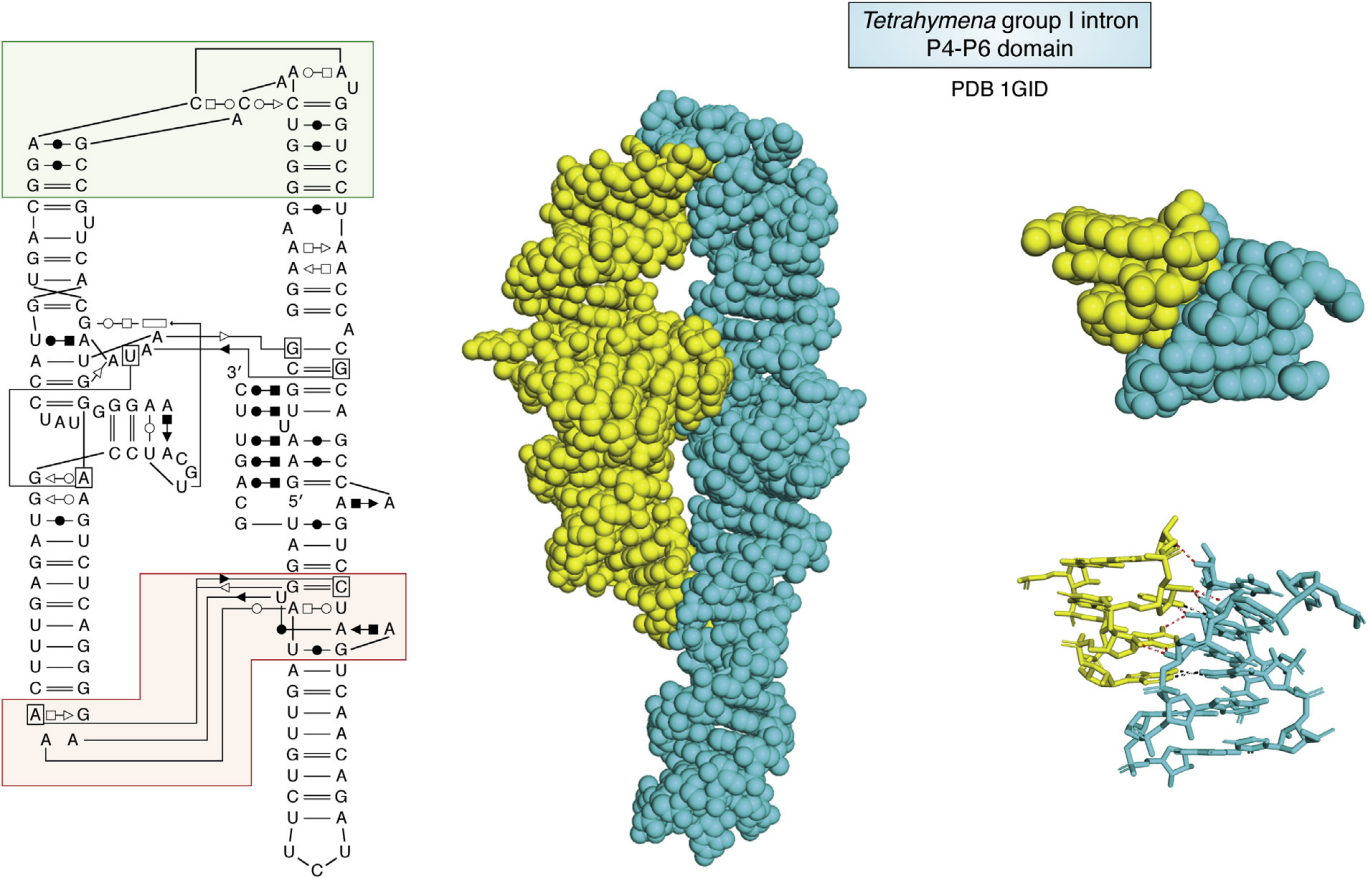
**From *left* to *right*, a two-dimensional view of the P4–P6 domain with the nomenclature described in**Figure 3***B*** (240) **and, with next to it, the whole molecule shown in space-filling mode highlighting the coaxial stacking of helices and their parallel packing.** In the *left* two-dimensional view, the region boxed in *green* shows how the RNA is able to bend 180° and the regions boxed in *red* are shown in space-filling and atomic views on the *right* of the figure; these views show the precise and tight contact with the tetraloop GAAA and its receptor, called the 11-nt motif; notice that there are twice as many H-bonds between the hydroxyl groups (*red dotted lines*) than between the bases (*black dotted lines*). That type of RNA–RNA contacts were discovered through sequence analysis and SELEX experiments (244).

The quest for the structure of the hammerhead ribozyme took more than 10 years before a coherent picture emerged and overall, about 20 years since its discovery (82, 83) (for short overviews see (84, 85)). In any case, it displays a three-way junction maintained in the proper relative orientations of the helices by non-Watson–Crick pairs (Fig. 5). As mentioned above, the structure of P4–P6, a fragment of the group I intron from *Tetrahymena thermophila* (65), unveiled several key recurrent RNA folding rules. As alluded to above, that structure, beyond the breathtaking views on RNA folds, initiated a strong impetus on the RNA crystallography community. An illustration is shown in Figure 6 and short descriptions in Table 1.

## Form and function

The quote from Wainwright (86) captures aptly the conundrum: “Structure without function is a corpse; function without structure is a ghost” (86). And it would take a lot more pages to do justice to what RNA structures have taught us about biology. Many RNA structures have been milestones and fueled our advances in understanding biochemical function and biological evolution. Forty years after the establishment by Tom Cech (87) and Sidney Altman (88) that all cells exploit RNA catalysis, we now have high-resolution structures of different catalytic RNAs in various stages (see Fig. 1) (89, 90, 91, 92, 93). We have also learned from ribosome structures that peptide bond catalysis during translation is performed in an RNA only environment (94, 95, 96). And the recent structures of the active complexes of the spliceosome (see Fig. 2) (97, 98, 99, 100), together with the structural similarities with the autocatalytic group II introns (101, 102), indicate clearly also that the chemistry of splicing is performed by RNA elements. In those large RNP complexes, such as the ribosomes and the spliceosomes, the RNA elements are positioned precisely for function by the concomitant actions of RNA folding and protein complex formation. In the ribosome field, technological development in cryo-electron microscopy has led to stunning increases in resolution; for example, we now can delve inside ribosomal structures at 2 Å resolution (103, 104). Further, active ribosomes are assembled through long and convoluted maturation processes. Structures are now appearing showing various steps in the maturation of ribosomes with changes in RNA base pairing and structures and with binding of maturation cofactors absent in the final assembled ribosomes (105, 106, 107, 108). While the ribosome performs the programmed steps in translation (initiation, translocation, termination) essentially as a dynamical single complex entity, this is not the case for the spliceosome. Driven by several splicing or regulatory factors and RNA-dependent ATPase/helicases, the RNA and protein composition as well as the structures of the spliceosomal complex change along the catalytic steps, from the recognition of the 5’ and 3’ splice junctions and the branching point leading to the B∗ complex ready for the first step of splicing (cleavage at 5’ splice site) to the C∗ complex ready for the second step of splicing, the ligation of the exons, and the disassembly of the spliceosome for initiating a new cycle (98, 99). As shown in Figure 2, we now can visualize many of the states along the spliceosomal cycle and derive illuminating movies after decades of sustained efforts by several groups around the world.

One should always keep in mind the famous quote by T. Dobzhansky: “Nothing in Biology Makes Sense except in the Light of Evolution” (109). The accumulated RNA structures offer us amazing insights into the principles of molecular evolution underlying biological evolution. Powerful techniques in molecular evolution have demonstrated that starting from a random sequence one can isolate sequences specific for binding a given ligand or with a defined function (110, 111, 112). The structures of many aptamers are in the PDB now and new ones are still being selected and crystallized, such as the RNA fluorophores (113, 114, 115). The SELEX experiments led to the discovery of riboswitches in bacteria (116). Riboswitches are RNA sequences that, during transcription in the presence of a given ligand, fold into a native structure different from the one obtained after transcription in the absence of the same ligand (117, 118, 119). The alternative folds exert different action on the production of metabolic enzymes through transcription termination (or antitermination) or inhibition (or not) of ribosome binding for initiation of translation. There are now many structures of riboswitches with and without ligand bound (117, 120). Amazingly, the ligand can be as small as a fluoride ion (121) or as large as vitamin B12 (122, 123). Furthermore, there are several families of sequences for the same or similar ligand; up to four classes of guanidine and SAM riboswitches have been identified (120, 124).

Beyond the comforting insights on the RNA world at the origins of life about 2.8 billion years ago, all these RNA structures, coupled with structural alignments of homologous sequences, offer us multiple “Rosetta stones” for deciphering the RNA molecular evolution principles (125). Prior to the visualization of three-dimensional RNA architectures, RNA sequence alignments played major roles (and they still continue to do so). Through the analysis of nucleotide positions covarying according to the Watson–Crick rules, one can deduce the secondary structure (*i.e.*, the ensemble of base-paired RNA helices) of a set of homologous RNA molecules. The third kingdom of life, the *Archaea*, were discovered by Carl Woese by sequencing ribosomal RNA and aligning them (126, 127). The bacteria are still identified according to the 16S rRNA sequences (128). The secondary structures of many functional structured RNAs were obtained by sequence alignments (129, 130, 131, 132, 133, 134). The iconic cloverleaf structure of all cellular tRNAs was deduced with the first two sequences determined (37, 38). The availability of both RNA sequences and RNA structures allows structural alignment patterned on RNA architectures. These allow us to learn about how RNAs evolve, in other words how changes or mutations in homologous sequences maintain the RNA folded architecture. One can learn which variations are neutral and which molecular interactions are conserved through species. When several homologous three-dimensional structures are available, one can learn also how molecular accommodations occur and how ions or water molecules compensate for the loss of some interaction. By analysis of sequence alignments coupled with structures, one can also visualize how molecular units can swap or interchange and determine which interactions are opportunistic and not critical for form and function. Integrated databases with sophisticated techniques for guaranteeing the interoperability of data will need to be developed to exploit fully the one-dimensional and the three-dimensional data. In complexes between RNA and proteins, the diversity and multiplicity of intermolecular contacts are enormously potentiated. The understanding of RNP formation and structure is a major challenge for years to come.

## A short history of RNA modeling and RNA assembly computational tools

In the 1980s, the PDB also accepted structures derived by computational modeling. One of us did deposit several structures of RNA modeled on the basis of sequence alignments and/or chemical and enzymatic probing in solution. While PDB may have accepted these submissions somewhat reluctantly, many of these modeled structures were of intense interest to the growing crowd of RNA aficionados, and their accessibility stimulated experimentalists to put them to the test. After presenting the modeling of the core of group I introns (135) at *The 49th Pittsburgh Diffraction Conference*, Columbus, in November 1991, there was almost a riot in the room with half of the participants arguing for and the other half against the validity of the procedure. These modeled structures can no longer be found on the PDB but can be retrieved from the following website https://eric-westhof.ibmc.cnrs.fr/. Interestingly, wwPDB recently held a workshop on data standards for integrative structural models (136), and *Protein Data Bank Japan* (PDBj) recently launched the *Biological Structure Model Archive* (BSM-Arc) (137).

**Figure 7:**
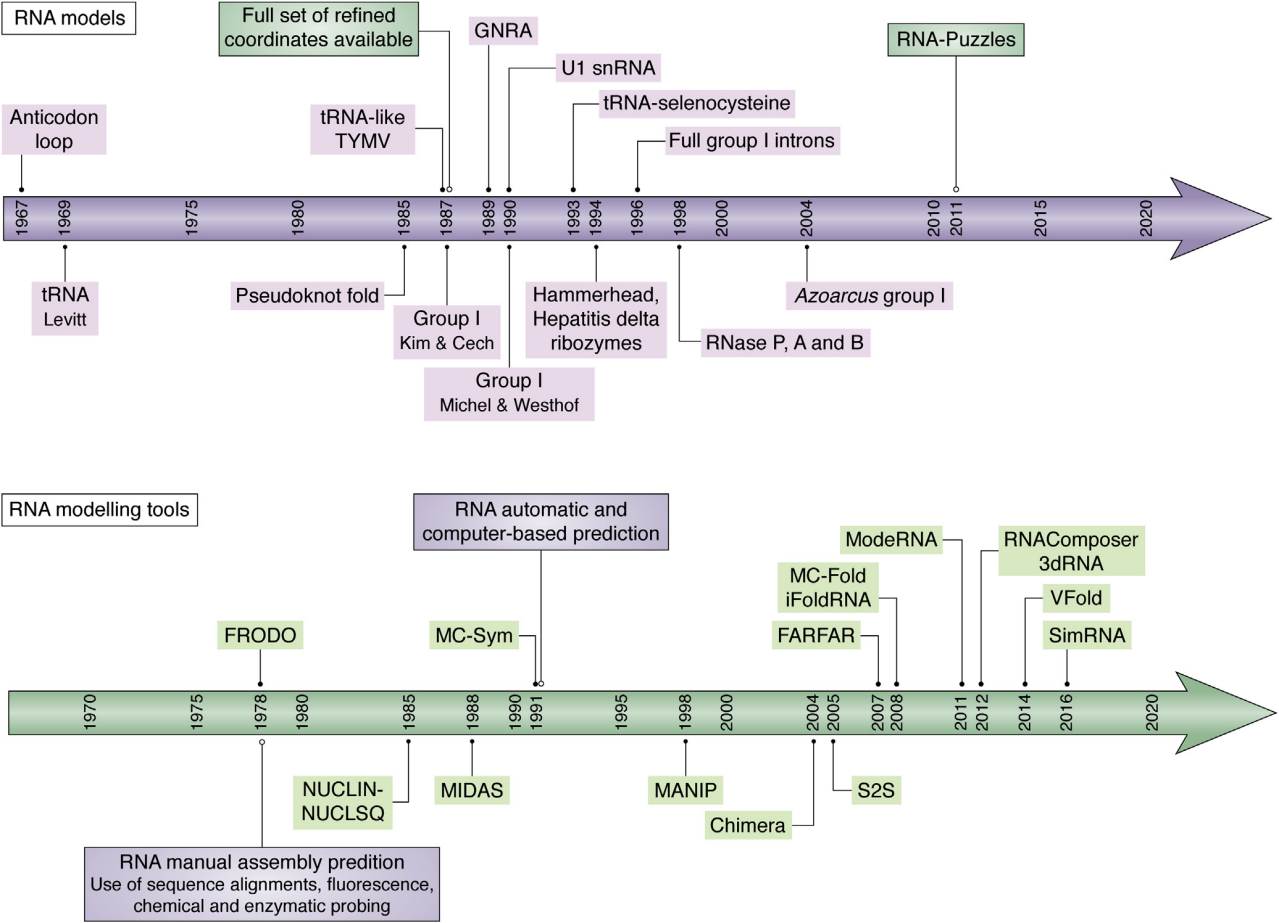
***Top*, a timeline of RNA models.** Before 1987, no sets of coordinates for the suggested models were made available. With the development of modeling tools based on computer graphics, one could derive coordinates (without manually building physical models) and refine them (see *bottom*). Along time, the following RNA models are highlighted: (1) the anticodon loop (138); (2) the Levitt tRNA (141); (3) the pseudoknot fold (143); (4) the Kim & Cech core of group I intron (144); (5) the tRNA-like in TYMV (245); (6) the GNRA loop in 5S rRNA (246); (7) the Michel & Westhof core of group I intron (135); (7) the 4-way junction of U1 snRNA (188); (8) the tRNA selenocysteine (247); (9) the hammerhead (248) and the hepatitis delta (249) ribozymes; (10) full group I introns (250); (11) A and B families of RNase P (251); (12) the *Azoarcus* group I intron (252). Many RNA structures were also modeled afterward, especially within the RNA-Puzzles Consortium (155, 156, 157, 158). *Bottom*, a timeline of some RNA assembly and computing tools. The most recent ones are regularly used and actively improved. (1) FRODO developed by Alwyn Jones was a pioneering tool in computer molecular graphics (151); (2) NUCLIN-NUCLSQ (159), an inclusive refinement program dedicated to nucleic acids and based on Hendrickson–Konnert PROLSQ (253, 254); (3) MIDAS (255, 256); (4) MC-Sym (257); (5) MANIP (258); (6) Chimera (259); (7) S2S (260, 261); (8) FARFAR (262); (9) MC-Fold (263), iFoldRNA (264); (10) ModeRNA (265); (11) RNAComposer (266); 3dRNA (267); (12) VFold (268); (13) SimRNA (269).

Figure 7 (top) shows a timeline of selected RNA models that have been published. It starts with the modeling work of Fuller and Hodgson (138) on the anticodon loop of tRNA. They manually assembled space filling (139) and Kendrew (140) models. They stated very clearly the objectives that are still of value today: “We do not necessarily believe that our models describe the actual molecular conformations to an accuracy of a few hundredths of an angstrom, but the analysis shows that a model with the general characteristics we propose can be built with acceptable stereochemistry. Only if model building is treated as a rigid discipline with strict attention paid to detailed stereochemistry can the results of a study such as this be considered reliable and meaningful.” They did identify and rationalize the correct stacking of the five nucleotides on the 3’-end of the loop with the 3’-strand of the anticodon helix (called 3’-stack). Michael Levitt assembled in 1969 a whole tRNA (141). For that model, the available sequences were used and analyzed. The choice of the stacked arms followed previous small-angle X-ray scattering (142) (Fig. 3, *A*–*C*). Interestingly, contacts between the D- and T-loops are proposed as well as base pairs between residues 8 and 14 as well as 15 and 48 (now sometimes called the Levitt pair), but both in the usual Watson–Crick configurations instead of the observed *trans* configuration (Fig. 4, *D* and *E*). Sequence analysis coupled with model building led to the suggestion that some RNAs can form pseudoknots, for example, between a hairpin loop and a single strand (meaning that the single strand does not go through the loop) (143). This fold, now on the logo of the International RNA Society (https://www.rnasociety.org/), is present in a large number of functional structured RNAs. In 1987, Kim and Cech (144) published a model of the core of group I intron on the basis of the available experimental data and with clear model building principles that are still valid. They are worth restating: “(i) RNA duplexes were assumed to have A-form RNA helix conformation. (ii) If two duplex stems were separated by fewer than three unpaired nucleotides, they were stacked collinearly. (iii) If one helix competed with two others for collinear stacking, the two helices separated by the least number of unpaired nucleotides were chosen to form a stacked helix. (iv) Non-Watson-Crick base-pairing was allowed at the junction of two helices. (v) Single “bulged” bases were stacked within a helix. (vi) If two conserved bases in a single-stranded region were in proximity, base-pairing was attempted subject to the constraints of the chemical-modification data. (vii) Loop conformations were taken from those in the tRNA structure.” Kim had built the tRNA structure in electron density in Alex Rich’s laboratory (145, 146) some years before. The coaxial stacks P3–P7–P8 and P4–P6 were identified (144). Afterward, with the development of molecular graphics and refinement programs, full molecular models with coordinates could be generated (Fig. 7).

Figure 7 (bottom) shows the evolution of the computer tools necessary for the manipulation and refinement of RNA structures. The advent of computer graphics and the development of systems allowing the display and the manipulation of molecular objects in real time, pioneered by Levinthal (147), Feldmann (148), Langridge (149, 150), and Jones (151), were breakthroughs in structural biology. Several of these tools were developed for crystallography and modeling purposes as well. Model building is the process in crystallography (or cryo-EM) in which one actually assembles a molecular model by fitting molecular objects into electron density (manually either with physical objects in a Richard’s box or with computer graphics, a process now almost automatically done). The model building is guided and constrained by the electron density. The molecular models are then refined geometrically and stereochemically so as to satisfy best the crystallographic data or the electron density. In *ab initio* modeling, the molecular objects are built without the constraints of crystallographic data. The model building can be driven by geometric and physicochemical energetic terms in (semi-) automatic fashion by various computing tools (for RNA, see the recent reviews (152, 153)). Such tools should integrate the knowledge, accumulated at a given time, on the molecular systems to be modeled. This knowledge integration is still a formidable challenge owing to the diversity of molecular interactions to consider and treat simultaneously (weak forces, water molecules, ions, etc.). When performed manually with physical objects, model building is still very powerful to assimilate and understand the rules underlying the folding of macromolecules. Richard Hamming said, “The purpose of computing is insight, not numbers.” Similarly, the purpose of modeling is insight, not models. One can also recall the famous quote from Richard Feynman: “What I cannot create, I do not understand.”

The point of archiving 3D models is to provide a record of previous modeling efforts that can help to benchmark methodological progress in computational modeling and to develop metrics to assess contemporary modeling efforts (see (154)). Toward that aim, the *RNA-Puzzles* computational contests were created in 2011 (155, 156, 157, 158). The *RNA-Puzzles* consortium is a community-wide assessment of RNA 3D structure prediction that aims to expose the bottlenecks in current RNA 3D structure prediction and promote the improvement of these prediction methods. RNA-Puzzles attempts to provide the RNA modeling community what CASP (*Critical Assessment of Methods of Protein Structure Prediction*) has been doing for protein modeling (159). All the data sets for the modeled structures and codes for assessment are now available as open source on GitHub (https://github.com/RNA-Puzzles) (160).

## Databases play central roles in science

In 2003, three organizations, the *Research Collaboratory for Structural Bioinformatics* (RCSB) in the United States, the *Macromolecular Structure Database* (MSD) at EBI, and the PDBj in Osaka, created the worldwide PDB (wwPDB; http://www.wwpdb.org/) with the goal of maintaining a single archive of structure data of macromolecules that is freely and publicly available to the global community (161). The Biological Magnetic Resonance Data Bank is now also part of wwPDB, and soon the *China Protein Data Bank* will join. This structure also facilitates unified deposition, curation, and distribution of structures to the growing global community of structural biologists and scientists that use 3D data (161). At the same time, each independent center is free to innovate new visualization and analysis tools to promote the reuse of the data in creative and useful ways for nonspecialists. Each center is also engaged in creating educational materials to make structural biology accessible to students at all levels, for example, PDB101’s *Molecule of the Month* and the PDBj’s *Encyclopedia of Protein Structures* (162). A number of the *Molecule of the Mo*nth entries are RNA or DNA structures, and several others are proteins that interact with nucleic acids.

We would like to recall here a sentence written, in 1990, by Brändén and Jones (163): “It is the crystallographer’s responsibility to make sure that incorrect protein structures do not reach the literature.” They could as well have written: “It is the structural biologist’s responsibility to make sure that incorrect macromolecular structures do not reach the literature,” which would encompass also NMR and cryo-EM technologies as well as nucleic acids and their complexes. PDB continues to adapt in the new century by adopting new data formats to accommodate the much larger structures being solved. The development of metrics for assessing structure validity both globally and locally at the nucleotide level is proving key to understanding the significance of structural variation and mapping out the dynamic behavior of nucleic acid structures in response to interactions with small molecules, proteins, and other nucleic acids. Cryo-EM is rapidly displacing X-ray crystallography for very large complexes and molecular machines that cycle through multiple functional states and are hard to crystallize in unique states. New metrics for cryo-EM are in progress and should soon be available (164). In this context, referees and journal editors have also a major role to play, to prevent incorrect structures from reaching the literature and worse, the archival databases. Referees are encouraged to request (and in many cases are actually provided with) complete and detailed statistics tables, validation reports and quality indicators, coordinates, and electron-density maps. Journal editors, as well as authors, should comply with such requests despite fierce competition for publication. The *PDB* has played an important role by providing sophisticated metrics and automation that provides rapid feedback to contributors of data when they submit structures.

Databases constitute an absolute necessity for advancing science, to facilitate the work of scientists in the same and related disciplines. They are repositories of observational data organized with carefully curated dictionaries and controlled vocabularies upon which future science can build and develop. In the immediate future, there are at least two main challenges to which we would like to draw attention. Owing to the sizes and complexities of modern databases, these two challenges are interconnected and interdependent.

First, the sustainability and maintenance of databases for the use of a now internationalized community of scientists raise the issue of how the associated costs shall be equitably apportioned; these are recurrent costs to support trained and competent personnel, together with the maintenance and constant upgrading of the extensive infrastructure required to keep up with the growth in the size and complexity of the data. Short-term grants with a dedicated focus are not adapted to maintain large international resources (164). New business models are being explored and promoted (165). The *Global Biodata Coalition* is an initiative started by the *International Human Frontier Science Program Organization* that according to their website (https://globalbiodata.org) aims “to stabilize and ensure sustainable financial support for the global biodata infrastructure and in particular to identify for prioritized long-term support a set of *Global Core Data Resources* that are crucial for sustaining the broader biodata infrastructure.”

And, secondly, databases fundamentally comprise well-organized archives of chosen sets of objects. In structural biology, the objects are mainly molecular sequences and structures. However, as these two types of data attach to the same natural entities, they need to be integrated to grasp biological function and evolution more holistically. New developments in cryo-electron tomography (166), which allows visualization of macromolecules *in situ*, will open up visualization of macromolecules in their cellular environment. Maybe one day we will also include digital descriptions of phenotypes, including bones, skeletons, or wings in order to relate their forms and colors to the underlying genes and genetic networks. Launched by the *RNAcentral Consortium* in 2014 at the *EMBL-EBI*, *Wellcome Genome Campus* (Hinxton, UK), *RNAcentral* (https://rnacentral.org) integrates and unifies access to all types of noncoding RNA sequences from all organisms. This is a major enterprise that goes in the right direction for the integration and interoperability of data. A bridge between sequences and standard secondary structures has been achieved recently (167). In the future this bridging integration should be extended to three-dimensional structures and thus the PDB and the NDB.

## In the light of evolution

It would take a whole book with several dozens of figures to convey the amazing knowledge accumulated on RNA structures and architectures since the beginning of RNA structural biology and the advent of the PDB. Only glimpses of RNA structural biology are presented here, and we would like to apologize for not mentioning the contributions of many structural biologists, especially those using NMR spectroscopy, a field that contributed several seminal structures, for example, on RNPs and telomerase (168, 169, 170, 171, 172), with unique critical data on RNA dynamics (173, 174, 175, 176, 177). Almost each RNA structure still brings some surprising and unexpected features, and these unveil the constraints of the physicochemical interactions and the congruent accommodations due to the historical contingencies of biological evolution. If we can cite again T. Dobzhansky (109): “Seen in the light of evolution, biology is, perhaps, intellectually the most satisfying and inspiring science. Without that light it becomes a pile of sundry facts, some of them interesting or curious but making no meaningful picture as a whole.” A quote from Ernest Rutherford states “All science is either physics or stamp collecting,” which is in sharp contrast to the lifelong accumulation of observations and data by the biologist Charles Darwin. The RNA structures in the PDB are absolutely not a pile of miscellaneous items, “sundry facts”; they are physical objects, which, together with genomic data, allow us to integrate present-day biological functions and the historical evolution in all living species on earth.

Technological improvements and breakthroughs are at the heart of discovery and scientific progress. One may recall the famous quote from Sydney Brenner “progress in science depends on new techniques, new discoveries and new ideas, probably in that order” (178). As alluded above, the recent and constant progress in cryo-EM techniques is totally changing structural biology (179). The protein prediction field is now overwhelmed by the achievements of artificial intelligence tools (180, 181, 182). For those raised with the warning of the Levinthal’s paradox (183), we admire in awe the progress and breakthroughs. Could such tools be applied to RNA structure prediction? Since they are based on the analysis of hundreds of structures, do we have enough RNA structures sufficiently nonredundant? As remarked by Jane Richardson (184), among the “1491 RNA-only structures, just 373 of which have a chain ≥60 nucleotides” and of those a large number consists of ribosomal structures with various ligands or from various biological sources. As appropriately reminded by John Helliwell (185), in the end, these breakthroughs would not have been feasible without the PDB.

## Dedication

**Figure:**
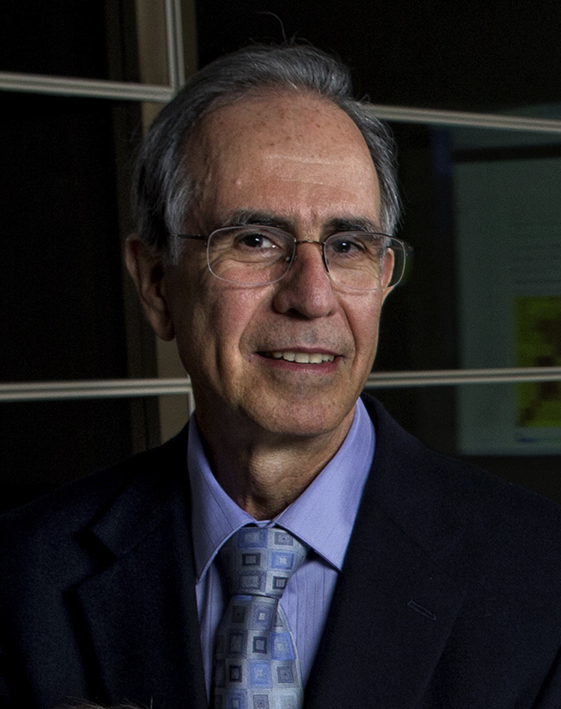
Neocles B. Leontis (1955–2020)

During the writing of this work, on December 8, 2020, **Neocles B. Leontis** died in a car accident (186). Together with the editors, we would like to dedicate this article to his memory. I have been fortunate to exchange and collaborate with Neocles for more than 20 years. Beyond the many scientific papers published together, always occasions of in-depth, dynamic, and always fair discussions, Neocles was a friend intensely human, joyous, and positive with whom it was recomforting to be. I admired him deeply for his move toward local politics (in 2019, he was elected to the City Council of Bowling Green, Ohio) and his involvement in many social and climate issues. He applied his intellectual power to these issues as thoroughly and seriously as he did to the various scientific problems he tackled. His untimely and brutal death is a loss to the local community around his University and town, but also to all the RNA scientists around the world who use the concepts, tools database he contributed to develop. Neocles earned his PhD from Yale with Peter Moore as mentor. He became professor of Chemistry at Bowling Green State University in 1987. With Peter Moore, he worked using NMR on the 5S rRNA and showed the key role of Mg ions and the presence of non-Watson–Crick pairs. Later using sequence comparisons, he could identify loop E modules in RNA. Together we published a nomenclature for classifying logically and nonambiguously non-Watson–Crick. Neocles Leontis and his group at Bowling Green developed many websites and tools for analyzing and compiling RNA structures.

## Conflict of interest

The authors declare that they have no conflicts of interest with the contents of this article.
